# Protective Effects of Sulforaphane on Exercise-Induced Organ Damage via Inducing Antioxidant Defense Responses

**DOI:** 10.3390/antiox9020136

**Published:** 2020-02-04

**Authors:** Ruheea Taskin Ruhee, Sihui Ma, Katsuhiko Suzuki

**Affiliations:** 1 Graduate School of Sport Sciences, Waseda University, Tokorozawa 359-1192, Japan; ruhee@fuji.waseda.jp; 2 Faculty of Sport Sciences, Waseda University, Tokorozawa 359-1192, Japan

**Keywords:** sulforaphane, inflammation, antioxidant, exercise, Nrf2, oxidative stress, cytokines, antioxidant enzymes

## Abstract

Regular exercise is beneficial to maintain a healthy lifestyle, but the beneficial effects are lost in the case of acute exhaustive exercise; this causes significant inflammation, oxidative stress along with organ damage. Recently, sulforaphane (SFN), an indirect antioxidant, has drawn special attention for its potential protective effect against inflammation and oxidative stress. However, no studies have been performed regarding acute exhaustive exercise-induced organ damage in association with SFN administration. Therefore, the aim of this study was to investigate the effects of SFN on acute exhaustive exercise-induced organ damage and the mechanisms involved. To perform the study, we divided mice into four groups: Control, SFN, exercise, and SFN plus exercise. The SFN group was administered orally (50 mg/kg body wt) 2 h before the running test. We measured plasma levels of alanine aminotransferase (ALT), aspartate aminotransferase (AST), and lactate dehydrogenase (LDH), and acute exhaustive exercise significantly increased these biomarkers. In addition, the mRNA expression of pro-inflammatory cytokines, IL-6, IL-1β, and TNF-α, were significantly increased in the liver of exercise group. However, the SFN plus exercise group showed a significant reduction in the expression of cytokines and blood biomarkers of tissue damage or cell death. Furthermore, we measured mRNA expression of Nrf2, heme oxygenase (HO)-1, and antioxidant defense enzymes expression, i.e., superoxide dismutase (SOD1), catalase (CAT), and glutathione peroxidase (GPx1) in the liver. The expression of all these biomarkers was significantly upregulated in the SFN plus exercise group. Collectively, SFN may protect the liver from exhaustive exercise-induced inflammation via inducing antioxidant defense response through the activation of Nrf2/HO-1 signal transduction pathway.

## 1. Introduction

Over the decades, an enormous amount of research regarding physical exercise and its effects on health has been carried out. Regular physical exercise is the utmost important tool to maintain a healthy lifestyle. It has both therapeutic and preventive effects against chronic diseases [1] such as obesity and its metabolic complications [2], type 2 diabetes [3], cardiovascular disease, hypertension [4], cancer [5], depression, anxiety, stress, dementia, etc. [6]. Regular physical exercise increases the production of reactive oxygen species (ROS). However, the response to this ROS depends on the intensity or load and duration of exercise [7]. Despite the individual variation, exercise load is further associated with physiological stress [8]. During exercise, oxygen demand is increased in the body by up to 15-fold, while for the active tissues it is increased by 100-fold (especially in muscle) in comparison to resting conditions [9]. Moreover, while we acutely do exercise, the energy demand is provided by hepatic glycogen, which is used as an energy source instead of fat oxidation. Therefore, liver glycogen is depleted rapidly, although training can reduce the rate of glycogen loss [10]. The liver is the main organ that metabolizes endogenous and exogenous compounds and detoxifies toxic ones to maintain regular homeostasis via the circulation. It is also a major source of ROS production after an exhaustive exercise [11]. The increased ROS production may alter other biological functions, as well as cause damage to extrahepatic organs [12].

Free radicals are constantly produced in our bodies at a certain level in different tissues via cellular respiration processes (as a part of normal metabolism). Excessive production of free radicals or ROS can further cause oxidative damage to cellular organelles such as mitochondria, and the macromolecules they contain. Endurance exercise is associated with a high degree of oxygen consumption and free radical damage, followed by an increased level of inflammation, which can be identified by circulating pro-inflammatory mediators such as interleukin (IL)-6, IL-1β, tumor necrosis factor (TNF)-α, and nitric oxide (NO) [13]. Collectively, this response is known as systemic inflammatory response syndrome (SIRS) [14]. Exercise also increases the inflammation via the accumulation of neutrophils and cytokines in the active tissues [15,16]. During high-intensity exercise, gut-epithelium permeability increases and endotoxins (i.e., lipopolysaccharide (LPS)) can easily leak into the portal circulation, where they are further transported to the liver. From the liver they can further pass to the central circulation [17]. LPS is a component of Gram-negative bacteria that triggers an inflammatory cascade by inducing the production of pro-inflammatory cytokines [18]. Thus, depending on intensity and duration, exercise can alter circulating endotoxin concentrations. In other words, LPS administration can also mimic the exercise-induced endotoxemia [19]. However, chronic oxidative stress or inflammation leads to the onset of degenerative diseases. In this regard, direct or indirect antioxidants may decrease free radicals to prevent chronic degenerative disease and maintain a healthy condition [20]. Naturally, all mammals have both enzymatic and nonenzymatic antioxidant defense systems to maintain the redox (oxidation-reduction) balance and prevent inflammation.

To date, research with nonnutritive substances derived from plant foods (further known as phytonutrients) is quickly advancing for the development of new remedies for various pathological conditions. These phytonutrients alter the immune system via several cellular signaling pathways [21,22]. Moreover, dietary phytonutrients can influence cellular processes associated with various diseases and conditions associated with increased stress. Naturally occurring isothiocyanates (IST) are enzymatically degraded products of glucosinolates (GST, the precursor of isothiocyanate), found abundantly in cruciferous vegetables, i.e., cauliflower, cabbage, broccoli, and brussels sprouts [23]. GST is water soluble but does not show biological activities directly. When plant cells are ruptured by food processing or damaged by microbial infection, the myrosinase enzyme hydrolyzes the inactive GST into active IST, thiocyanates, and nitriles [23,24].

Sulforaphane (SFN) is the most studied naturally occurring IST, a hydrolysis product of glucoraphanin, predominantly found in broccoli. However, cooked broccoli contains no myrosinase, but gut microbiota can convert GST to SFN, as they possess myrosinase-like activity [25]. SFN is a promising diet-derived IST, also the most potent inducer of phase 2 antioxidant enzymes (heme oxygenase-1 (HO-1), NAD(P)H: Quinone oxidoreductase type 1 (NQO1), glutathione S-transferase, UDP-glucuronosyltransferase (UGT), and sulfotransferase (SULT)). These enzymes mainly participate in the detoxification process of toxic molecules or ROS through conjugation reactions, and protect against cellular and peripheral damage, as well as reduces the risk of cancer and other degenerative diseases attributed to oxidative stress or inflammation [26,27]. The redox sensitive transcription factor nuclear factor E2 factor-related factor (Nrf2) plays a pivotal role in stimulating the activation of antioxidant response elements (ARE). Nrf2, which belongs to the cap “n” collar (CNC) family of basic region-leucine zipper (bZIP) transcription factors, is the key factor regulating the antioxidant response [28]. Nrf2 activity can be regulated by dietary phytochemicals and other chemoprotective agents [29]. In the homeostatic condition, the activity of Nrf2 signaling pathway remains suppressed in the cytoplasm by the repressor protein Keap1 (Kelch-like erythroid cell-derived protein with CNC homology-associated protein 1), localized near the plasma membrane, which targets Nrf2 for proteasomal degradation [30]. Furthermore, in response to the pathological condition associated with oxidative damage by ROS, Nrf2 readily translocates into the nucleus and interacts with specific ARE, followed by stimulating phase 2 enzyme gene expression [31,32,33].

The electron transport chain (ETC) in the mitochondria is the major site of ROS production. Naturally endogenous antioxidant enzymes superoxide dismutase (SOD), catalase (CAT), and glutathione peroxidase (GPx) are the first-line defense system, while the integrated action of these enzymes resists redox disturbance in the cells [34]. Glutathione (GSH) plays a crucial role in the antioxidant defense system, demonstrating unique antioxidant properties. The liver is the major site for GSH synthesis (90% of circulating GSH is synthesized in the liver) and under oxidative stress it is mobilized to other organs through the circulation [35]. Reduced GSH is utilized by GPx to H_2_O_2_ and converted to oxidized GSH and water. This reaction is important for the multicomponent defense system within the cells [36].

In this study, we examined the effect of SFN on exercise-induced inflammation, as well as pro-inflammatory cytokine gene expression in the liver. This includes a mechanism involving a signal transduction pathway via alteration of antioxidant enzyme gene expression by potential induction of Nrf2 and its dependent phase 2 detoxification enzyme HO-1. Nrf2 activation by SFN was previously observed in a cell culture study (in vitro) [36] and in this report we detail whether these effects can be reproduced in the body (in vivo).

## 2. Materials and Methods

### 2.1. Animals

Thirty-six male C57BL/6 mice (seven weeks old) were purchased from the Takasugi Experimental Animals Supply (Kasukabe, Japan) and three to four mice were caged together (27 × 17 × 13 cm) under a temperature-controlled environment for two weeks with a 12:12 h light-dark cycle (08:00 to 20:00 light period and 20:00 to 08:00 was dark period). All the mice had free access to a standard chow diet (MF Oriental Yeast, Tokyo, Japan) and water until the experiment started. Mice were divided into four groups: Two for sedentary; control (CON, *n* = 9), SFN (*n* = 9), and two for exercise (Ex, *n* = 9) and SFN + Ex (*n* = 9). The experimental procedures followed the Guiding Principles for the Care and Use of Animals in the Academic Research Ethical Review Committee of Waseda University and were approved (2019-A109).

### 2.2. Exhaustive Exercise Protocol

**Figure 1:**
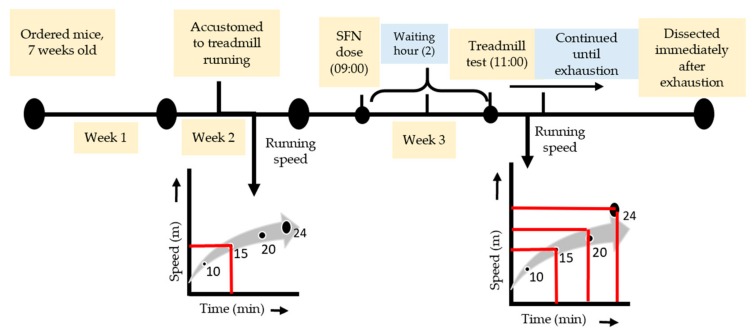
Experimental design and exhaustive exercise protocol.

One week before the exhaustive running exercise, all mice (including sedentary) were accustomed to a motorized treadmill (Natsume, Kyoto, Japan) running at 15 m/min for 10 min (0% incline). SFN (LKT Laboratories, St. Paul, MN, USA, product code S8044) was administered orally (50 mg/kg body wt), solubilized in distilled water with a volume of 150 µL on the day of the experiment at around 9 am. The uphill treadmill running (7% incline) was started 2 h after feeding. The dose and waiting hours were selected based on the previous literature [37,38]. The Ex and SFN + Ex groups of mice were subjected to run at 15 m/min for 10 min, followed by 20 m/min for 10 min, and finally, 24 m/min continued until exhaustion (Figure 1). The state of exhaustion was defined as the inability to continue running despite tapping on the back of the mouse several times or repositioning by hand on the treadmill [39,40].

### 2.3. Sample Collection

The sedentary groups were sacrificed 2 h after oral ingestion and the exercise group was sacrificed immediately after exhaustion under light anesthesia with the inhalant isoflurane (Abbott, Tokyo, Japan). Blood samples were collected rapidly from the abdominal aorta using heparin under inhalant mild anesthesia, and centrifuged at 1500× *g* for 15 min at 4 °C to collect the plasma. The liver sample was immediately collected and stored in liquid nitrogen, and all the other samples were stored at −80 °C.

### 2.4. Plasma Biochemical Parameters

Plasma levels of alanine aminotransferase (ALT), aspartate aminotransferase (AST), and lactate dehydrogenase (LDH) were measured by Koutou-Biken Co. (Tsukuba, Japan).

### 2.5. Real-Time (RT) Quantitative Polymerase Chain Reaction (qPCR)

Total RNA was extracted from the liver using the TRizol (Thermo Fisher, Rockford, IL, USA) extraction reagent, according to the maker’s protocol. The NanoDrop system (NanoDrop Technologies, Wilmington, DE, USA) was used to confirm the concentration and purity of extracted RNA by the ratio of A260/280. Total RNA was reverse transcribed to cDNA using the High-Capacity cDNA Reverse Transcription kit (Applied Biosystems, Foster City, CA, USA) according to the provided instructions. PCR was performed using the Fast 7500 real-time PCR system (Applied Biosystems, Foster City, CA, USA) and Fast SYBR Green PCR Master Mix (Applied Biosystems, Foster City, CA, USA). The thermal profile for all genes consisted of one denaturing cycle at 95 °C for 10 min, 40 cycles consisting of denaturing at 95 °C for 3 s and annealing and elongation at 60 °C for 15 min. 18S mRNA was used as the housekeeping gene. All data were normalized using the ΔΔCT method and stated as a fold change relative to the values of the control group. Specific sequences of primers used for gene amplification are given in Table 1.

### 2.6. Statistical Analysis

Data are expressed as the mean ± standard error (SE). A two-way analysis of variance (ANOVA) was performed to identify the main effect of SFN administration and/or exercise. To determine the significant difference among means, Tukey’s post-hoc test was performed. Statistical significance was defined as *p* < 0.05, using IBM SPSS, V 25.0 (IBM Corp., Armonk, NY, USA).

## 3. Results

### 3.1. Effect of SFN on Plasma Levels of Exercise-Induced AST, ALT, and LDH

**Figure 2:**
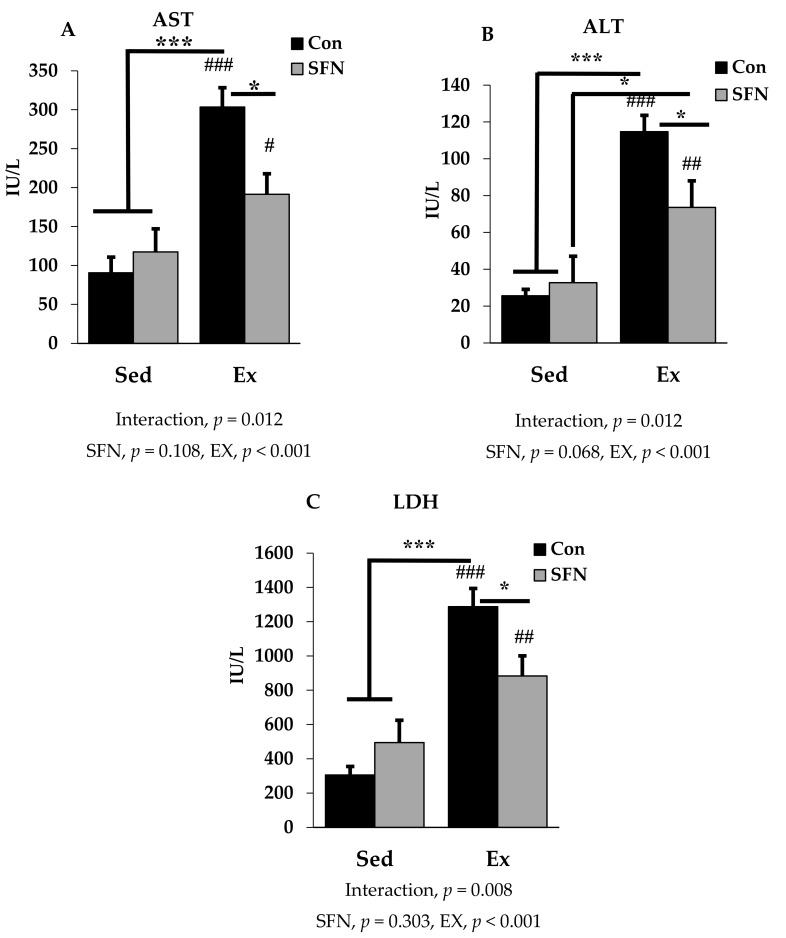
Plasma enzyme level comparison between the sedentary (Sed) and exercise (Ex) groups, with (sulforaphane; SFN) or without (control; Con) supplement. Figure 2 (**A**–**C**) represent aspartate aminotransferase (AST); alanine aminotransferase (ALT) and lactate dehydrogenase (LDH) levels in plasma. ### *p* < 0.001, ## *p* < 0.01, # *p* < 0.05 as compared to the sedentary control. *** *p* < 0.001, * *p* < 0.05. Interaction of SFN and Ex written as “Interaction”. Data are presented as mean ± SE.

Figure 2A–C presents plasma AST, ALT, and LDH levels among the sedentary and exercise groups in the absence or presence of SFN. AST or glutamic oxaloacetic transaminase (GOT), and ALT or glutamic pyruvic transaminase (GPT) are two important markers for hepatocellular damage. Exercise with or without SFN treatment significantly increased AST, ALT, and LDH levels in the plasma, compared with the control and/or sedentary groups (*p* < 0.001). However, there was a significantly less induction in all three biomarkers in the Ex + SFN group compared to the exercise group alone (*p* < 0.05). LDH is an important marker, that increases during prolonged exercise [39,41]. In addition to that, measuring AST and ALT, and assessing LDH gives a clear diagnosis of liver injury.

### 3.2. Effect of SFN on Exercise-Induced Pro-Inflammatory Cytokine Expression

**Figure 3:**
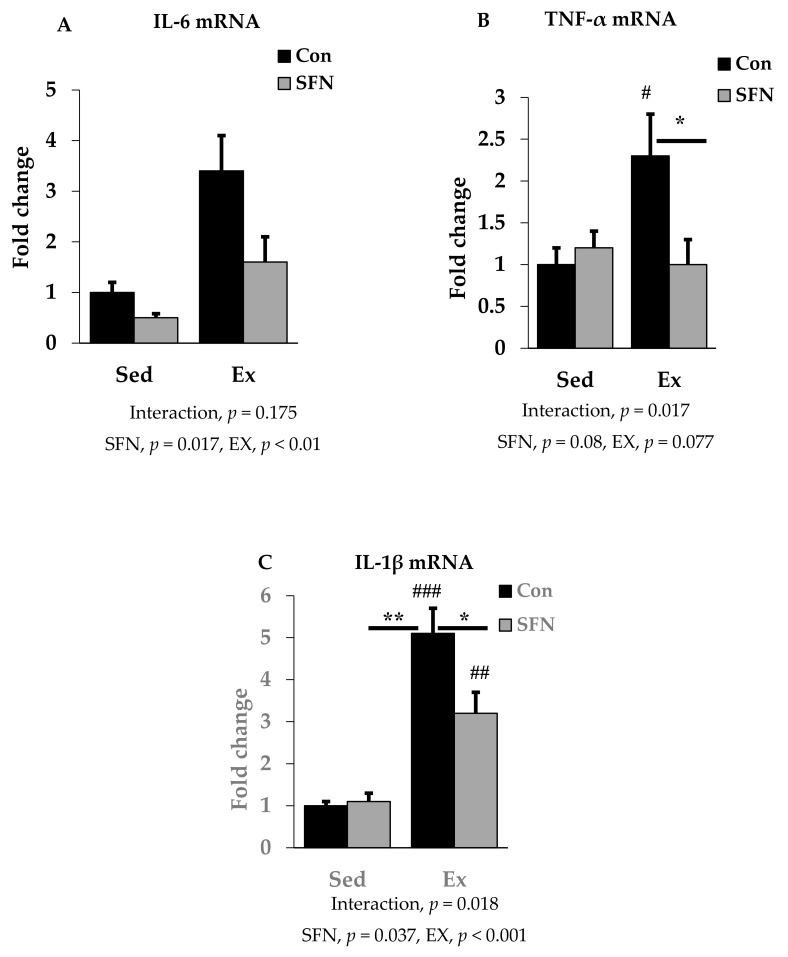
Interleukin-6 (IL-6) mRNA (**A**), Tumor necrosis factor-α (TNF-α) mRNA (**B**), and Interleukin-1β (IL-1β) mRNA (**C**) expression in the comparison between the sedentary (Sed) and exercise (Ex) groups, with (sulforaphane; SFN) or without (control; Con) supplement. ### *p* < 0.001, ## *p* < 0.01, # *p* < 0.05 as compared to the sedentary control. ** *p* < 0.01, * *p* < 0.05. Interaction of SFN and Ex written as “Interaction”. Data are presented as mean ± SE.

We analyzed the mRNA expression of pro-inflammatory cytokines IL-6, TNF-α, and IL-1β using RT-qPCR in the liver to identify the effect of SFN on pro-inflammatory cytokine levels after the endurance running exercise. Results are expressed as the relative differences from sedentary control as the baseline. While IL-6 mRNA expression was increased by 3.4-fold after exercise, the Ex + SFN group showed less increase in the mRNA expression (1.6-fold) (Figure 3A); however, the interaction was not significant. Figure 3B,C showed results for TNF-α and IL-1β mRNA expression levels. The exercise group showed increased mRNA expression by 2.3- and 5.1-fold, respectively, while the Ex + SFN group showed less expression, showing changes of 1- and 3.2-fold, respectively.

### 3.3. Effect of SFN on Antioxidant Defense System Gene Expression

**Figure 4:**
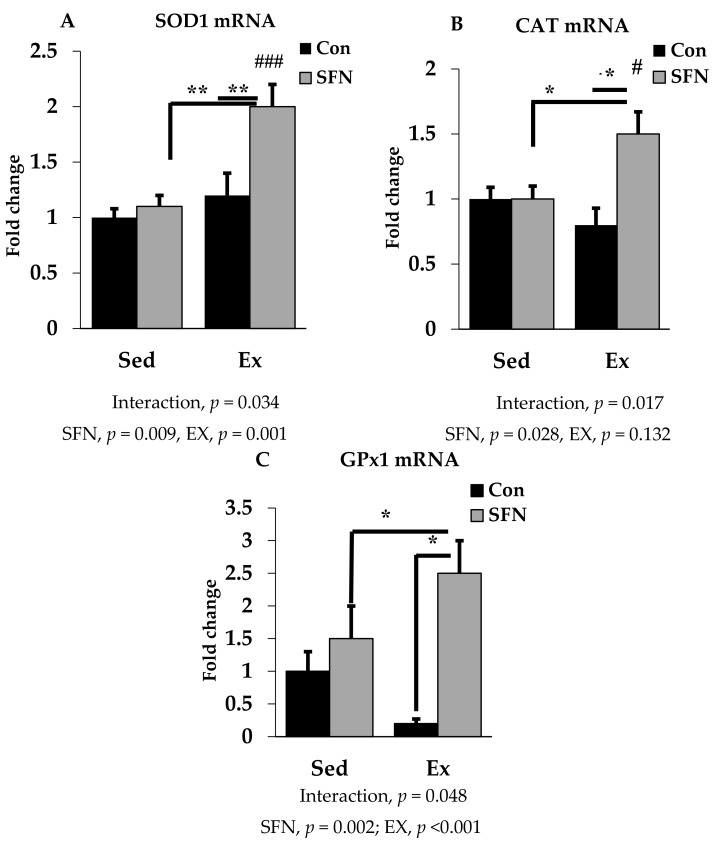
Superoxide dismutase 1 (SOD1) mRNA (**A**), Catalase (CAT) mRNA (**B**), and Glutathione peroxidase 1 (GPx1) mRNA (**C**) expression in the liver in the comparison between the sedentary (Sed) and exercise (Ex) groups, with (sulforaphane; SFN) or without (control; Con) supplement. ### *p* < 0.001, # *p* < 0.05 as compared to the sedentary control. ** *p* < 0.01, * *p* < 0.05. Interaction of SFN and Ex written as “Interaction”. Data are presented as mean ± SE.

SOD1, CAT, and GPx1 are the prime endogenous defense enzymes in the redox balance system against ROS. As shown in Figure 4A–C, the mRNA expression of SOD1, CAT, and GPx1 enzymes was significantly upregulated by 2-, 1.5-, and 2.5-fold with exercise in the SFN group. A slightly reduced expression was observed only in the exercise group for CAT and GPx1, but not for SOD1. However, no significant difference was observed in the level of those enzymes between the exercise and control groups.

### 3.4. Effect of SFN on Nrf2/HO-1 Expression

**Figure 5:**
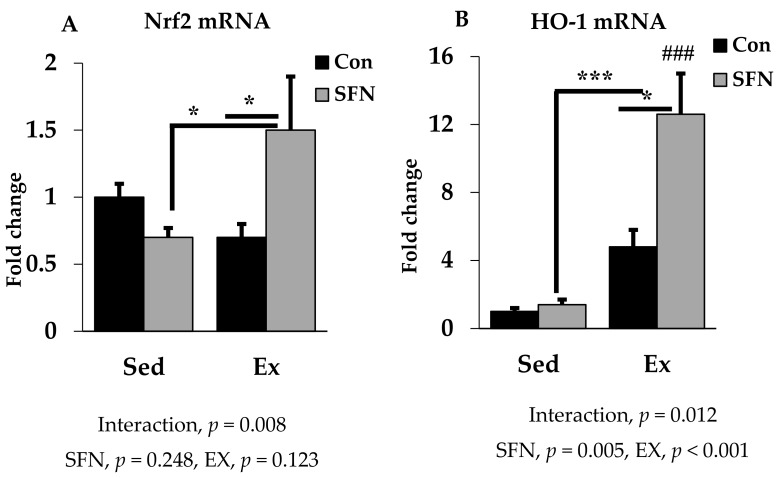
Nuclear factor E2 factor-related factor (Nrf2) mRNA (**A**) and Heme oxygenase 1 (HO-1) mRNA (**B**) expression in the liver in the comparison between the sedentary (Sed) and exercise (Ex) groups, with (sulforaphane; SFN) or without (control; Con) supplement. ### *p* < 0.001 as compared to the sedentary control. *** *p* < 0.001, * *p* < 0.05. Interaction of SFN and Ex written as “Interaction”. Data are presented as mean ± SE.

Nrf2 is the pivotal transcription factor for regulating the cellular antioxidant system. In Figure 5A, Nrf2 mRNA expression was increased by 1.5-fold with SFN in the exercise group, which differs significantly as compared to the exercise and/or sedentary SFN groups. Interestingly, as shown in Figure 5B, phase 2 enzyme HO-1 gene expression was increased by 12.6-fold in the Ex + SFN group, which is around a three-times larger expression than the nonsupplement exercise group. In addition, the interaction effect of both expression levels was significant (*p* < 0.05).

## 4. Discussion

Here, we investigated the protective effect of SFN treatment, which induces the antioxidant defense system through the Nrf2/HO-1 signaling pathway, on acute exhaustive exercise-induced inflammation. Previously, we have conducted an in vitro study looking at the anti-inflammatory role of SFN (using LPS to mimic exercise-induced inflammation) by inducing the Nrf2/HO-1 signal transduction pathway [19]. This study was designed to confirm the signal transduction mechanisms in vivo responsible for inducing gene expression of pro-inflammatory cytokines and its attenuation via altering the mRNA expression of Nrf2/HO-1 and endogenous antioxidant defense enzymes. However, no study was found describing the effect of SFN administration on exercise-induced organ damage via inducing this signal transduction mechanism. To the best of our knowledge, this is the first report analyzing the protective effect of SFN on exercise-induced liver damage. Therefore, as an initial screen we analyzed different organ damage markers in the plasma and found significant values for the presented data.

Exhaustive exercise can improve our neural functions and learning behavior [42]. However, acute exhaustive exercise can accelerate oxidative stress and inflammation significantly due to the increased oxygen consumption. This can further downregulate the expression of endogenous antioxidant defense mechanisms [43]. From past research, controversial evidence was found, regarding antioxidant supplementation that modulates endurance capacity and prevents tissue damage [39,40,44,45,46,47]. As a natural antioxidant, SFN has drawn great attention because of its potential contribution to chemo-preventive activities through multiple signaling pathways [48]. Despite knowing that SFN is a very strong inducer of phase 2 enzymes, very little is known about the protective effect of SFN on acute exercise-induced inflammation or organ damage.

Cellular damage biomarkers can be identified in the plasma after a single bout of exhaustive exercise [14,16,49,50]. Different enzymes are released into the bloodstream due to tissue damage in several organs such as liver, kidney, or muscle. AST and ALT are commonly used as pathological damage markers for liver tissue, and the elevated levels of these markers represent loss of the cell membrane’s functional integrity [51]. Acute exercise causes significant injury in the liver, increasing the expression of these enzymes [52]. Our study shows a consistently increased level in the nonsupplement exercise group, while the SFN with exercise group showed a reduced level of AST and ALT. Previous studies have reported that pretreatment with SFN attenuates liver injury by reducing serum levels of AST, ALT, and LDH through the Nrf2/ARE pathway [53,54]. A recent study by Lee et al. indicated that SFN may suppress lethal hepatic injury by modulating inflammatory pathways and may contribute to the treatment of liver diseases [55]. In addition to that, LDH in the blood is a sensitive indicator for both liver and muscle damage, and also sometimes for different types of cancer [56]. In the sedentary SFN group, all the markers (AST, ALT, and LDH) were slightly increased in comparison to control, but the difference between groups was not significant. We presume that without exercise, chronic administration of SFN might cause hepatotoxicity. In addition, the effect of brief stimulation of Nrf2 signaling pathway by SFN also provides long-lasting protection from radical-induced diseases [57]. Furthermore, creatinine kinase (CK) is another useful marker to identify exercise-induced injury. Elevated levels of both CK and LDH after exercise are considered as markers of muscle injury; however, an elevated level of LDH and normal CK level is considered as a liver injury marker [58].

Pro-inflammatory cytokines and chemokines that are released into the circulation after a single bout of exercise, cause substantial damage to the liver [59,60]. Among the cytokines, IL-6 is a multifunctional one that exerts both pro- and anti-inflammatory effects, depending on classic or trans-signaling modes [61]. TNF-α and IL-1β are vital mediators that can regulate acute phase protein (APP) synthesis, also responsible for acute hepatic inflammation [62,63]. The regulatory factor nuclear factor kappa-light-chain-enhancer of B cells (NF-κB) can be activated after a single bout of exercise [64]. In response to exercise, circulating pro-inflammatory cytokines activate the inhibitor of NF-κB (IκB) kinase complex (IKK). After activation, NF-κB with its attached subunits p65 and p50 translocate into the nucleus from the cytoplasm through the phosphorylation of IκB, and induces pro-inflammatory gene expression [65]. Antioxidant supplementation can suppress the inflammatory response and attenuate the expression of circulating pro-inflammatory cytokines (IL-6, IL-1β, and TNF-α) [66,67]. SFN administration can suppress the expression of the NF-κB signaling pathway and via inducing the Nrf2 signal transduction pathway [68], can reduce mRNA expression of IL-6 and IL-1β in liver tissue [69]. In our study, pro-inflammatory cytokine (IL-6, IL-1β, and TNF-α) expression in the exercise group was increased significantly in the liver tissue, while the SFN-supplemented exercise group showed reduced expression of these pro-inflammatory cytokines. Nrf2 signal activation has also been reported to downregulate the overproduction of pro-inflammatory cytokines [70]. In our previous study, we found that SFN can prevent LPS-induced inflammation through the Nrf2/HO-1 signaling pathway [19]. Since endotoxins pass into the systemic circulation via the liver, we assume that SFN is a promising compound for the downregulation of pro-inflammatory cytokines circulating in the liver during high-intensity exercise.

The antioxidant enzymes, SOD1, CAT, and GPx1 are actively involved in reducing ROS. Previous research activities supported that a single bout of acute exhaustive exercise dramatically increases significant production of ROS from activated neutrophils, thereby suppressing antioxidant enzymes activity, i.e., GPx1, SOD1, and CAT in the liver tissue, which further impairs the free radical scavenging system [71,72,73]. In this study, we analyzed antioxidant enzyme (cytosolic SOD, CAT, and cytosolic and mitochondrial GPx1) mRNA expression in the liver tissue to identify whether SFN administration has any effect on these enzymes during exhaustive exercise. Previous studies have shown that SFN pretreatment can upregulate the SOD and GPx enzyme activities to reduce oxidative stress, which is associated with the Nrf2/HO-1 signaling pathway [53]. After exhaustive exercise, in the liver, the elevated xanthine oxidase (XO) activity is responsible for excessive production of superoxide radical [74]. Here, we found that SFN supplementation in the exercise group increases expression of SOD and GPx1 enzymes. Decreased CAT and GPx level might make liver more susceptible to oxidative stress [74]. However, our data show that SFN administration can alter the expression of these enzymes, thereby protecting liver from oxidative stress. Another study reported that treatment with SFN can significantly increase mRNA expression of SOD1 and CAT and other Nrf2 target genes to protect cells from oxidative stress [75]. Based on previous records, antioxidant enzyme expression increases in muscle after exhaustive exercise rather than liver or kidney [40,76]. Therefore, antioxidant supplementation or a combination of several major antioxidants may attenuate exercise-induced ROS production and contribute to endurance capacity [77]. Additional evidences from other studies have shown that multiple doses of SFN have a significant protective effect against muscle damage through the activation of Nrf2-dependent antioxidant/detoxification phase 2 enzymes [78,79]. Moreover, providing a SFN-enriched diet for 45 days, reduced exhaustive exercise-induced oxidative damage of the liver by increasing antioxidant enzymatic activities [80].

Nrf2 is an important transcription factor that binds to ARE regions in the promoters of genes during stressed conditions and induces antioxidant defense enzymes and phase 2 enzymes in tissues [81,82]. Indeed, transcriptional upregulation of Nrf2 inhibits pro-inflammatory cytokine expression [19,83]. Application of phytochemicals holding anti-inflammatory or anti-oxidative properties can trigger Nrf2 activation [70], also enhancing endurance capacity [78]. Our results are consistent with the previous studies that Nrf2 gene expression in the liver tissue was upregulated in the exercise group with SFN, which upregulated the phase 2 enzyme HO-1. Furthermore, multiple Nrf2-induced genes can be activated by treatment with SFN [84]. SFN can readily undergo a conjugation reaction in the liver after absorption [85], consequently inducing phase 2 enzyme and antioxidant enzyme expression via the Nrf2 signaling pathway. This mechanism could explain the protective role of SFN in exercise-induced inflammation and oxidative stress via the signal transduction mechanism of Nrf2/HO-1 activation.

Our study has several limitations; we measured gene expression of selected cytokines and enzymes, since we aimed to report on the mechanism of signal transduction. Although there are multiple published data on cell culture studies, SFN may regulate phase 2 detoxification and antioxidant enzyme expression; however very little is known about the in vivo studies. Therefore, we performed a single dose oral administration of SFN and presented findings on the protective effect of SFN on acute exhaustive exercise-induced inflammation, without directly measuring ROS levels. Our results also support that a single dose of SFN may protect liver damage by inducing phase 2 antioxidant enzyme expression via Nrf2 activation; however, SFN treatment showed no effect on the endurance capacity between the exercise groups. The dose we used in the present study (50 mg/kg body weight SFN), was a level not easily obtained by consuming an SFN-rich food. Further investigations are required to determine whether a similar effect may be observed through consuming SFN-rich foods.

## 5. Conclusions

Natural antioxidants have unique scavenging properties against free radicals. Much research has been carried out, regarding antioxidant supplementation to modulate exercise-induced stress and inflammation. Despite the somewhat paradoxical effects, exercise is important for our longevity and for living a sound life. Here, we presented data on the protective role of SFN on acute exercise-induced liver damage. Collectively, we summarized that SFN is not directly involved in the ROS reduction process, but it induces a cellular defense system through the induction of antioxidant enzymes via the upregulation of the Nrf2/HO-1 signal transduction pathway, thereby reducing oxidative stress and inflammation.

## Figures and Tables

**Table 1:** Specific primer sequences for RT-qPCR.

Target Gene	Accession Number (Size, bp)	Forward (5′ → 3′)	Reverse (5′ → 3′)
*18S*	NM_011296 (127)	TTCTGGCCAACGGTCTAGACAAC	CCAGTGGTCTTGGTGTGCTGA
*IL-6*	NM_031168 (76)	TAGTCCTTCCTACCCCAATTTCC	TTGGTCCTTAGCCACTCCTTC
*TNF-α*	NM_013693.1 (102)	CCTCCCTCTCATCAGTTCTA	ACTTGGTGGTTTGCTACGAC
*IL-1β*	NM_008361.4 (183)	GGGCCTCAAAGGAAAGAATC	TTGCTTGGGATCCACACTCT
*SOD1*	GI:56270594 (1107)	GAGACCTGGGCAATGTGACT	GTTTACTGCGCAATCCCAAT
*CAT*	XM_028766902 (100)	CAGAGAGCGGATTCCTGAGAGA	CTTTGCCTTGGAGTATCTGGTGAT
*GPx*	NM_001329527.1 (751)	AGTACGGATTCCACGTTTGA	GGAACTTCTCAAAGTTCCAG
*Nrf2*	NM_010902(100)	GAGTCGCTTGCCCTGGATATC	TCATGGCTGCCTCCAGAGAA
*HO-1*	NM_010442 (175)	CACGCATATACCCGCTACCT	CCAGAGTGTTCATTCGAGCA

## References

[1] Suzuki K. (2019). Chronic inflammation as an immunological abnormality and effectiveness of exercise. Biomolecules.

[2] Bouchard C., Depres J.P., Tremblay A. (1993). Exercise and obesity. Obes. Res..

[3] Thomas D., Elliott E.J., Naughton G.A. (2006). Exercise for type 2 diabetes mellitus. Cochrane Database Syst. Rev..

[4] Painter P.L., Hector L., Ray K., Lynes L., Paul S.M., Dodd M., Tomlanovich S.L., Ascher N.L. (2003). Effects of exercise training on coronary heart disease risk factors in renal transplant recipients. Am. J. Kidney Dis..

[5] Newton R.U., Galvao D.A. (2008). Exercise in prevention and management of cancer. Curr. Treat. Options Oncol..

[6] Pedersen B.K., Saltin B. (2015). Exercise as medicine–evidence for prescribing exercise as therapy in 26 different chronic diseases. Scand. J. Med. Sci. Sports.

[7] Wenger H.A., Bell G.J. (1986). The interactions of intensity, frequency and duration of exercise training in altering cardiorespiratory fitness. Sports Med..

[8] Lambert M.I., Borresen J. (2010). Measuring training load in sports. Int. J. Sports Physiol. Perform..

[9] Cooper C., Vollaard N.B., Choueiri T., Wilson M. (2002). Exercise, free radicals and oxidative stress. Biochem. Soc. Trans..

[10] Baldwin K., Fitts R.H., Booth F., Winder W., Holloszy J. (1975). Depletion of muscle and liver glycogen during exercise. Pflügers. Arch..

[11] Yoon M.Y., Kim S.N., Kim Y.C. (1997). Potentiation of acetaminophen hepatotoxicity by acute physical exercise in rats. Res. Commun. Mol. Pathol. Pharm..

[12] Sanchez-Valle V.C., Chavez-Tapia N., Uribe M., Mendez-Sanchez N. (2012). Role of oxidative stress and molecular changes in liver fibrosis: A review. Curr. Med. Chem..

[13] Suzuki K., Totsuka M., Nakaji S., Yamada M., Kudoh S., Liu Q., Sugawara K., Yamaya K., Sato K. (1999). Endurance exercise causes interaction among stress hormones, cytokines, neutrophil dynamics, and muscle damage. J. Appl. Physiol..

[14] Suzuki K., Nakaji S., Yamada M., Liu Q., Kurakake S., Okamura N., Kumae T., Umeda T., Sugawara K. (2003). Impact of a competitive marathon race on systemic cytokine and neutrophil responses. Med. Sci. Sports Exerc..

[15] Suzuki K. (2018). Cytokine response to exercise and its modulation. Antioxidants.

[16] Huang Q., Ma S., Tominaga T., Suzuki K., Liu C. (2018). An 8-Week, Low carbohydrate, high fat, ketogenic diet enhanced exhaustive exercise capacity in mice Part 2: Effect on fatigue recovery, post-exercise biomarkers and anti-oxidation capacity. Nutrients.

[17] Lim C.L., Suzuki K. (2017). Systemic inflammation mediates the effects of endotoxemia in the mechanisms of heat stroke. Biol. Med..

[18] Hung Y.-L., Suzuki K. (2017). The pattern recognition receptors and lipopolysaccharides (LPS)-induced systemic inflammation. Int. J. Res. Stud. Med. Health Sci..

[19] Ruhee R.T., Ma S., Suzuki K. (2019). Sulforaphane protects cells against lipopolysaccharide-stimulated inflammation in murine macrophages. Antioxidants.

[20] Mittler R. (2002). Oxidative stress, antioxidants and stress tolerance. Trends Plant Sci..

[21] Batchu S., Chaudhary K., Wiebe G., Seubert J. (2013). Bioactive food as dietary interventions for cardiovascular disease: Bioactive foods in chronic disease states. Bioactive Compounds in Heart Disease.

[22] Rao B.N. (2003). Bioactive phytochemicals in Indian foods and their potential in health promotion and disease prevention. Asia Pac. J. Clin. Nutr..

[23] Tang L., Paonessa J.D., Zhang Y., Ambrosone C.B., McCann S.E. (2013). Total isothiocyanate yield from raw cruciferous vegetables commonly consumed in the United States. J. Funct. Foods.

[24] Jeffery E.H., Stewart K.E. (2004). Upregulation of quinone reductase by glucosinolate hydrolysis products from dietary broccoli. Methods in Enzymology.

[25] Angelino D., Dosz E.B., Sun J., Hoeflinger J.L., Van Tassell M.L., Chen P., Harnly J.M., Miller M.J., Jeffery E.H. (2015). Myrosinase-dependent and–independent formation and control of isothiocyanate products of glucosinolate hydrolysis. Front. Plant Sci..

[26] Sheweita S., Tilmisany A. (2003). Cancer and phase II drug-metabolizing enzymes. Curr. Drug Metab..

[27] Zhang Y. (2011). Phase II Enzymes. Encycl. Cancer.

[28] Dhakshinamoorthy S., Jaiswal A.K. (2001). Functional characterization and role of INrf2 in antioxidant response element-mediated expression and antioxidant induction of NAD(P)H:quinone oxidoreductase1 gene. Oncogene.

[29] Stefanson A., Bakovic M. (2014). Dietary regulation of Keap1/Nrf2/ARE pathway: focus on plant-derived compounds and trace minerals. Nutrients.

[30] Velichkova M., Hasson T. (2005). Keap1 regulates the oxidation-sensitive shuttling of Nrf2 into and out of the nucleus via a Crm1-dependent nuclear export mechanism. Mol. Cell. Biol..

[31] Osburn W.O., Wakabayashi N., Misra V., Nilles T., Biswal S., Trush M.A., Kensler T.W. (2006). Nrf2 regulates an adaptive response protecting against oxidative damage following diquat-mediated formation of superoxide anion. Arch. Biochem. Biophys..

[32] Kobayashi A., Kang M.-I., Watai Y., Tong K.I., Shibata T., Uchida K., Yamamoto M. (2006). Oxidative and electrophilic stresses activate Nrf2 through inhibition of ubiquitination activity of Keap1. Mol. Cell. Biol..

[33] Itoh K., Tong K.I., Yamamoto M. (2004). Molecular mechanism activating Nrf2-Keap1 pathway in regulation of adaptive response to electrophiles. Free Radic. Biol. Med..

[34] Ighodaro O., Akinloye O. (2018). First line defence antioxidants-superoxide dismutase (SOD), catalase (CAT) and glutathione peroxidase (GPX): Their fundamental role in the entire antioxidant defence grid. Alexan. J. Med..

[35] Halliwell B., Gutteridge J. (2015). Antioxidant defences synthesized in vivo. Free Radicals in Biology and Medicine.

[36] Gathwala G., Aggarwal R. (2016). Selenium supplementation for the preterm Indian neonate. Indian J. Public Health.

[37] Jones S.B., Brooks J.D. (2006). Modest induction of phase 2 enzyme activity in the F-344 rat prostate. Bmc Cancer.

[38] Watson R.R., Preedy V.R. (2012). Dietary bioactive functional polyphenols in chronic lung diseases. Bioactive Food as Dietary Interventions for Liver and Gastrointestinal Disease.

[39] Yada K., Suzuki K., Oginome N., Ma S., Fukuda Y., Iida A., Radak Z. (2018). Single Dose administration of taheebo polyphenol enhances endurance capacity in mice. Sci. Rep..

[40] Yada K., Roberts L.A., Oginome N., Suzuki K. (2019). Effect of Acacia Polyphenol Supplementation on Exercise-Induced Oxidative Stress in Mice Liver and Skeletal Muscle. Antioxidants.

[41] Todd J.J. (2014). Lactate: Valuable for physical performance and maintenance of brain function during exercise. Biosci. Horiz..

[42] Radak Z., Suzuki K., Higuchi M., Balogh L., Boldogh I., Koltai E. (2016). Physical exercise, reactive oxygen species and neuroprotection. Free Radic. Biol. Med..

[43] Powers S.K., Ji L.L., Leeuwenburgh C. (1999). Exercise training-induced alterations in skeletal muscle antioxidant capacity: A brief review. Med. Sci. Sports Exerc..

[44] Packer L., Gohil K., DeLumen B., Terblanche S. (1986). A comparative study on the effects of ascorbic acid deficiency and supplementation on endurance and mitochondrial oxidative capacities in various tissues of the guinea pig. Comp. Biochem. Physiol. B.

[45] Kanter M., Nolte L., Holloszy J. (1993). Effects of an antioxidant vitamin mixture on lipid peroxidation at rest and postexercise. J. Appl. Physiol..

[46] Gohil K., Packer L., De Lumen B., Brooks G., Terblanche S. (1986). Vitamin E deficiency and vitamin C supplements: exercise and mitochondrial oxidation. J. Appl. Physiol..

[47] Sumida S., Tanaka K., Kitao H., Nakadomo F. (1989). Exercise-induced lipid peroxidation and leakage of enzymes before and after vitamin E supplementation. Int. J. Biochem..

[48] Juge N., Mithen R., Traka M. (2007). Molecular basis for chemoprevention by sulforaphane: a comprehensive review. Cell. Mol. Life Sci..

[49] Noakes T.D. (1987). Effect of exercise on serum enzyme activities in humans. Sports Med..

[50] Ma S., Suzuki K. (2019). Keto-adaptation and endurance exercise capacity, fatigue recovery, and exercise-induced muscle and organ damage prevention: A narrative review. Sports.

[51] Choudhary A.K., Devi R.S. (2014). Serum biochemical responses under oxidative stress of aspartame in wistar albino rats. Asian Pac. J. Trop. Dis..

[52] Ma S., Huang Q., Yada K., Liu C., Suzuki K. (2018). An 8-week ketogenic low carbohydrate, high fat diet enhanced exhaustive exercise capacity in mice. Nutrients.

[53] Zhao H.-D., Zhang F., Shen G., Li Y.-B., Li Y.-H., Jing H.-R., Ma L.-F., Yao J.-H., Tian X.-F. (2010). Sulforaphane protects liver injury induced by intestinal ischemia reperfusion through Nrf2-ARE pathway. World J. Gastroenterol..

[54] Liu W., XU Z.-f., GUO M.-x., LI H.-p., YANG T.-y., FENG S., XU B., DENG Y. (2016). Activation of Nrf2 pathway by sulforaphane against mercury-induced liver oxidative damage in rats. J. Env. Health.

[55] Lee C., Yang S., Lee B.-S., Jeong S.Y., Kim K.-M., Ku S.-K., Bae J.-S. (2019). Hepatic protective effects of sulforaphane through the modulation of inflammatory pathways. J. Asian Nat. Prod. Res..

[56] Jurisic V., Radenkovic S., Konjevic G. (2015). The actual role of LDH as tumor marker, biochemical and clinical aspects. Advances in Cancer Biomarkers.

[57] Bergstrom P., Andersson H.C., Gao Y., Karlsson J.-O., Nodin C., Anderson M.F., Nilsson M., Hammarsten O. (2011). Repeated transient sulforaphane stimulation in astrocytes leads to prolonged Nrf2-mediated gene expression and protection from superoxide-induced damage. Neuropharmacology.

[58] Khan H.A., Alhomida A.S., Sobki S.H., Habib S.S., Al Aseri Z., Khan A.A., Al Moghairi A. (2013). Serum markers of tissue damage and oxidative stress in patients with acute myocardial infarction. Biomed. Res..

[59] Feagins L.A., Flores A., Arriens C., Park C., Crook T., Reimold A., Brown G. (2015). Nonalcoholic fatty liver disease: A potential consequence of tumor necrosis factor-inhibitor therapy. Eur. J. Gastroenterol. Hepatol..

[60] Feng Y., Wang N., Ye X., Li H., Feng Y., Cheung F., Nagamatsu T. (2011). Hepatoprotective effect and its possible mechanism of Coptidis rhizoma aqueous extract on carbon tetrachloride-induced chronic liver hepatotoxicity in rats. J. Ethnopharmacol..

[61] Scheller J., Chalaris A., Schmidt-Arras D., Rose-John S. (2011). The pro-and anti-inflammatory properties of the cytokine interleukin-6. Biochim. Biophys. Acta..

[62] Tilg H. (2001). Cytokines and liver diseases. Can. J. Gastroenterol..

[63] Lacour S., Gautier J.-C., Pallardy M., Roberts R. (2005). Cytokines as potential biomarkers of liver toxicity. Cancer Biomark..

[64] Ji L., Gomez-Cabrera M., Steinhafel N., Vina J. (2004). Acute exercise activates nuclear factor (NF)-κB signaling pathway in rat skeletal muscle. Faseb J..

[65] Mittal M., Siddiqui M.R., Tran K., Reddy S.P., Malik A.B. (2014). Reactive oxygen species in inflammation and tissue injury. Antioxid. Redox Signal..

[66] Vassilakopoulos T., Karatza M.-H., Katsaounou P., Kollintza A., Zakynthinos S., Roussos C. (2003). Antioxidants attenuate the plasma cytokine response to exercise in humans. J. Appl. Physiol..

[67] Palmer H.J., Paulson K.E. (1997). Reactive oxygen species and antioxidants in signal transduction and gene expression. Nutr. Rev..

[68] Sun C.-C., Li S.-J., Yang C.-L., Xue R.-L., Xi Y.-Y., Wang L., Zhao Q.-L., Li D.-J. (2015). Sulforaphane attenuates muscle inflammation in dystrophin-deficient Mdx mice via Nrf2-mediated inhibition of NF-κB signaling pathway. J. Biol. Chem..

[69] Townsend B.E., Johnson R.W. (2017). Sulforaphane reduces lipopolysaccharide-induced proinflammatory markers in hippocampus and liver but does not improve sickness behavior. Nutr. Neurosci..

[70] Kim J., Cha Y.-N., Surh Y.-J. (2010). A protective role of nuclear factor-erythroid 2-related factor-2 (Nrf2) in inflammatory disorders. Mutat. Res..

[71] Peake J., Suzuki K. (2004). Neutrophil activation, antioxidant supplements and exercise-induced oxidative stress. Exerc. Immunol. Rev..

[72] Taysi S., Oztasan N., Efe H., Polat M., Gumustekin K., Siktar E., Canakci E., Akcay F., Dane S., Gul M. (2008). Endurance training attenuates the oxidative stress due to acute exhaustive exercise in rat liver. Acta Physiol. Hung..

[73] Korivi M., Hou C.-W., Huang C.-Y., Lee S.-D., Hsu M.-F., Yu S.-H., Chen C.-Y., Liu Y.-Y., Kuo C.-H. (2012). Ginsenoside-Rg1 protects the liver against exhaustive exercise-induced oxidative stress in rats. Evid-Based Complement. Altern. Med..

[74] Polavarapu R., Spitz D.R., Sim J.E., Follansbee M.H., Oberley L.W., Rahemtulla A., Nanji A.A. (1998). Increased lipid peroxidation and impaired antioxidant enzyme function is associated with pathological liver injury in experimental alcoholic liver disease in rats fed diets high in corn oil and fish oil. Hepatology.

[75] Chen X., Liu J., Chen S.Y. (2013). Sulforaphane protects against ethanol-induced oxidative stress and apoptosis in neural crest cells by the induction of Nrf2-mediated antioxidant response. Br. J. Pharm..

[76] Huang C.-C., Lin T.-J., Lu Y.-F., Chen C.-C., Huang C.-Y., Lin W.-T. (2009). Protective effects of L-arginine supplementation against exhaustive exercise-induced oxidative stress in young rat tissues. Chin. J. Physiol..

[77] Sen C.K. (2001). Antioxidants in exercise nutrition. Sports Med..

[78] Oh S., Komine S., Warabi E., Akiyama K., Ishii A., Ishige K., Mizokami Y., Kuga K., Horie M., Miwa Y. (2017). Nuclear factor (erythroid derived 2)-like 2 activation increases exercise endurance capacity via redox modulation in skeletal muscles. Sci. Rep..

[79] Malaguti M., Angeloni C., Garatachea N., Baldini M., Leoncini E., Collado P.S., Teti G., Falconi M., Gonzalez-Gallego J., Hrelia S. (2009). Sulforaphane treatment protects skeletal muscle against damage induced by exhaustive exercise in rats. J. Appl. Physiol..

[80] Cardenia V., Rodriguez-Estrada M.T., Lorenzini A., Bandini E., Angeloni C., Hrelia S., Malaguti M. (2017). Effect of broccoli extract enriched diet on liver cholesterol oxidation in rats subjected to exhaustive exercise. J. Steroid Biochem. Mol. Biol..

[81] Itoh K., Chiba T., Takahashi S., Ishii T., Igarashi K., Katoh Y., Oyake T., Hayashi N., Satoh K., Hatayama I. (1997). An Nrf2/small Maf heterodimer mediates the induction of phase II detoxifying enzyme genes through antioxidant response elements. Biochem. Biophys. Res. Commun..

[82] Kalayarasan S., Prabhu P.N., Sriram N., Manikandan R., Arumugam M., Sudhandiran G. (2009). Diallyl sulfide enhances antioxidants and inhibits inflammation through the activation of Nrf2 against gentamicin-induced nephrotoxicity in Wistar rats. Eur. J. Pharm..

[83] Lin W., Wu R.T., Wu T., Khor T.-O., Wang H., Kong A.-N. (2008). Sulforaphane suppressed LPS-induced inflammation in mouse peritoneal macrophages through Nrf2 dependent pathway. Biochem. Pharm..

[84] Hu R., Hebbar V., Kim B.-R., Chen C., Winnik B., Buckley B., Soteropoulos P., Tolias P., Hart R.P., Kong A.-N.T. (2004). In vivo pharmacokinetics and regulation of gene expression profiles by isothiocyanate sulforaphane in the rat. J. Pharm. Exp..

[85] University O.S. L.P.I. Isothiocyanates. https://lpi.oregonstate.edu/mic/dietary-factors/phytochemicals/isothiocyanates.

